# Electron microscopic changes of detrusor in benign enlargement of prostate and its clinical correlation

**DOI:** 10.1590/S1677-5538.IBJU.2016.0350

**Published:** 2017

**Authors:** Sher Singh Yadav, Rohit Bhattar, Lokesh Sharma, Gautam Banga, Trilok Chandra Sadasukhi

**Affiliations:** 1 Department of Urology and Renal Transplantation, SMS Medical College, Jaipur, Rajasthan, India; 2 Department of Urology, NIMS Medical College, Jaipur, Rajasthan, India; 3 SCI International Hospital, New Delhi, India; 4 Department of Urology, Mahatma Gandhi Hospital, Jaipur, Rajasthan, India

**Keywords:** Prostatic Hyperplasia, Prostate, Urinary Bladder

## Abstract

**Aims:**

To study the ultra structural changes in bladder musculature in cases of BPE and their clinical relevance.

**Material and Methods:**

In this descriptive longitudinal, controlled, observational study patients were enrolled into three groups, group 1, group 2A and group 2B. Control group (group-1) consisted of age matched normal male patients, who underwent surveillance or diagnostic cystoscopy for microscopic hematuria or irritative symptoms. Case group (group-2) comprised of patients with BPE, undergoing TURP. Case group (group-2) was further classified into: Category 2A (patients not on catheter) and category 2B (patients on catheter). All relevant clinical parameters like IPSS, prostate size, Qmax, PVR were recorded. Cystoscopy and bladder biopsy were performed in all patients. Various ultrastructural parameters like myocytes, fascicular pattern, interstitial tissue, nerve hypertrophy and cell junction pattern were analyzed under electron microscope and they were clinically correlated using appropriate statistical tests.

**Results:**

Control group had significant difference as compared to case group in terms of baseline parameters like IPSS, flow rate and prostate size, both preoperatively and postoperatively, except for PVR, which was seen only preoperatively. There was statistically significant difference in ultrastructural patterns between case and control group in all five electron microscopic patterns. However, no significant difference was found between the subcategories of case groups.

**Conclusions:**

BPE is responsible for ultra structural changes in detrusor muscle and these changes remain persistent even after TURP. Nerve hypertrophy, which was not thoroughly discussed in previous studies, is also one of the salient feature of this study.

## INTRODUCTION

Bladder dysfunction is often seen secondary to outlet obstruction in benign prostatic enlargement (BPE). These dysfunctions persist even after surgical correction and may be responsible for persistence of symptoms. However, the underlying mechanism for bladder dysfunction is not well understood. In clinical practice, urodynamic studies (UDS) can be used effectively to assess bladder function and degree of resistance, but its value in predicting the outcome of surgery has certain limitations. Earlier studies were done on animals to understand histological changes in bladder in cases of BPE (1), however, only little work had been done in this field on human beings. Aim of this study was to study electron microscopic changes in bladder muscle in cases of BPE and its clinical correlation.

**Figure 1:**
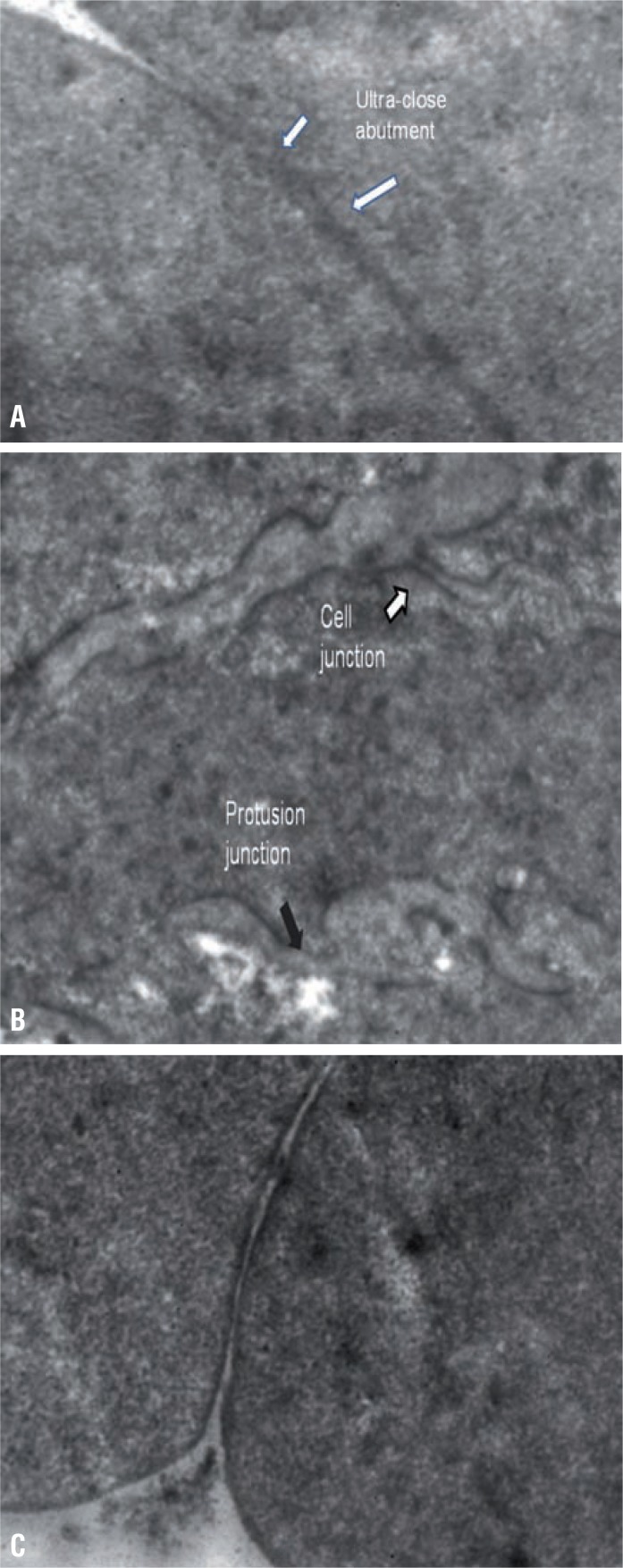
a) variant of normal Intercellular Junctions (x-16400); 1b) Myocytes with normal intercellular junction and protrusion junction (X-17000); 1c) normal Intercellular Junction (X-17600) this consists of two closely apposed 25-70nm wide gap sarcolemma lying parallel to each other for a length of up to 10μm with paired symmetrical dense plaques.

Normal bladder muscles are composed of fascicles. Fascicles in turn are made up of uni-directionally arranged four to twelve spindle shaped myocytes which are surrounded by interstitial microsepta, made up of collagen and occasionally by elastin (2). Hailemarium and Elbadawi graded fascicles as 1) Compact- Bundle of fascicles with occasional myocyte separation, 2) Intermediate- Mixture of compact and loose fascicles and 3) Loose- Moderate to severe myocyte separation or irregular arrangement with rarely seen uniform units (2). Together these muscle fascicles are compact and form muscle bundle and these too are also separated by collagen and elastin. Normal amount of collagen help in mechanical cell coupling, which help in complete bladder emptying. Collagen content in detrusor muscle is much varied and most studies are qualitative in nature, however Mirore et al. had showed that mean collagen content in normal detrusor muscle is <21% (3). For contraction, only a small proportion of myocytes are directly stimulated by nerve while majority of them receive the signals either through electrical or mechanical coupling via Intercellular junction (ICJ) that is why sparse axon bundles are seen in interstitium. Commonest ICJ is intermediate cell junction, which consists of two closely apposed cell wall (sarcolemma) lying parallel to each other for a length of up to 10 micro meters with paired symmetrical dense plaques. Other junction patterns are less frequently seen in normal detrusor. When myocytes are tightly opposed than that is known as “gap junction”. Other variants are “protrusion junction”, which are slender finger like projection between cells with tip to tip contact and “ultra close abutment” (Figure-1a), which are a tight opposition on a parallel surface in a shadow bomb impression configuration (4).

Although gap junctions are seen in normal detrusor, their ratio compared to normal ICJs increases in patients with detrusor instability, demonstrating a syncytium pattern of gaps between cell processes linking up to or more than ten myocytes. This leads to the summated detrusor contraction. So instead a low resistance pathway occur, thus mediating rapid electrical coupling. This ultimately results in the unstable contractions seen on urodynamic studies of subjects with an overactive detrusor (4, 5).

In pathological conditions, myocytes, interstitium and cell junction may show certain changes (6–8), these changes may be isolated or in various combinations. In BPE there may be changes in myocytes cell density, shape and content. Besides hypertrophy, myocytes may be empty or contain vacuoles and debris, their shape may be shriveled or disruptive. In dysfunctional bladder, myocytes can be breaded, branched, intertwined or bizarre shaped (9). In pathological conditions, fascicles may show marked separated arrangement. Abnormal fascicle arrangement and architecture is usually associated with abnormal interstitial tissue. Interstitium may have excessive collagen or elastin, loose fascicular pattern is more particularly associated with increased interstitial tissue and seen in hypocontractile bladder (6–8). When nerve is thickened over its length then it can be considered as hypertrophy. Although no study had exactly quantify this, in our study we considered >10 micrometer diameter as an abnormal finding.

## MATERIAL AND METHODS

After institutional review board approval the descriptive type of observational study with control group and longitudinal design was conducted in our department. Informed written consent was taken from all the patients. Patients attending the treatment of lower urinary tract symptom (LUTS), retention of urine and hematuria were enrolled. Detailed history was noted and physical examination was done in all patients. International prostate symptom score (IPSS) was recorded in all catheter free patients. Besides routine investigations, prostate specific antigen (PSA) estimation, and ultrasonography (USG) of kidneys, bladder and prostatic regions were also done in all the patients. Uroflowmetry (UFM) and post void residual urine (PVR) estimation were carried out in all the catheter free patients, whereas, UDS was also done in selected patients only. Patients with significant LUTS or retention of urine undergoing trans urethral resection of prostate (TURP) were enrolled in the case group (group-2). These patients were categorized into groups.

Control (group-1) comprised of age matched patients, who underwent cystoscopy for evaluation of microscopic hematuria or irritative LUTS without any evidence of BPE. Patients having IPSS >8, prostate volume>25mL, PVR >50mL, or peak flow rate (Qmax) <15mL/sec were excluded from this group.

Case (group-2) comprised of patients suffering from BPE. Only patients who had prostate volume >35mL with either retention of urine or having IPSS >15 and undergone TURP were included in this group. Patient having Qmax >15mL/sec or showing malignancy on TURP biopsy were excluded from this group. This group was further subcategorized into two groups: group 2A (catheter free) and group 2B (patients on catheter drainage).

Patients with past history of prostatic or bladder surgery, stricture urethra, neurological disorder, pelvic irradiation, prostatic/bladder malignancy, diabetes, renal impairment, prostatic or bladder abnormalities, active urinary tract infection, PSA>4ng/mL or on medical treatment (alfa blockers, 5 alfa reductase inhibitors, phospho diesterase 5 inhibitors {PDE 5I}, anticholinergics and cholinergics) and those with follow-up duration of less than 3 months, were also excluded from the study.

All patients underwent cystoscopy as standard procedure under anesthesia and findings were recorded. Bladder biopsy was taken using cold cup biopsy forceps. Minimum of two biopsies were taken, approximately 2cm supero-lateral to the ureteric orifices (2, 4). Following bladder biopsy, patients in case group underwent standard TURP. Biopsy specimens were immediately fixed in chilled buffered 4% formaldehyde solution and kept refrigerated at 5 degrees C until it processed to the concerned electron microscopic histopathology department. After that, mucosa was carefully stripped and detrusor muscle was separated. Later on, by using standard techniques, staining and fixation done and bladder biopsy specimen were examined under electron microscope. Electron microscopic histological features were evaluated regarding myocyte changes like degenerative pattern, fascicular arrangement, interstitial tissue pattern, nerve hypertrophy and communication between myocytes and these findings were compared with patient's clinical findings. Collagen content >34%, nerve diameter >10 micrometer and in ICJ, gap junction ratio >50% (gap junction/normal ICJ) were labeled as abnormal parameter in our study (3, 5).

Post operatively at the end of 1st and 3rd month patients of both groups were assessed by IPSS, PVR, Qmax and prostatic volume. Endoscopy and UDS were performed in selected cases.

In statistical analysis, continuous variables were summarized as mean and standard deviation, while categorical/nominal variables as proportions (%). One way ANOVA test with Post Hoc Tukey HSD test were used for analysis of continuous variables where subgroups were more than two, while chi—square test and Fisher exact test were used for nominal/categorical variables as per their indications. P value <0.05 was taken as significant. SPSS 21.0 version was used for all statistical calculation.

## RESULTS

In our patient series 50 patients met the inclusion criteria, with 21 patients in control group and 29 patients in case group. Case group 2A had 20 patients and group 2B had 9 patients. Table-1 shows the preoperative clinical parameters and comparison between various groups. Group-1 and group-2 were comparable in terms of their age but had statistically significant difference with respect to IPSS, flow rate and prostate size. In terms of PVR all three categories (group 1, group 2A and group 2B) had statistically significant differences.

**Table 1:** Preoperative (Baseline) comparison between groups.

Characters	Group	N	Mean	Std. Deviation	‘p’ Value*	Significant difference from#
Age(in years)	Control (Group 1)	21	60.19	3.49		
	Case (Group2 A)	20	62.35	5.70	0.289*	
	Case (Group2 B)	9	62.56	5.66		
IPSS	Control (Group 1)	21	3.14	1.46		
	Case (Group2 A)	20	20.45	3.90	<0.001	
	Case (Group2 B)	9	-+	-+		
Flow Rate(Qmax)	Control (Group 1)	21	17.29	2.62		A,B
	Case (Group2 A)	20	6.54	1.31	<0.001	1,B
	Case (Group2 B)	9	0.00+	0.00+		1,A
Prostate Size (in grams)	Control (Group 1)	21	16.00	2.35		A,B
	Case (Group2 A)	20	59.15	11.85	<0.001	1
	Case (Group2 B)	9	62.00	15.28		1
PVR(in mL)	Control (Group 1)	21	5.29	6.48		A,B
	Case (Group2 A)	20	88.35	50.14	<0.001	1,B
	Case (Group2 B)	9	400.00	242.38		1,A

* ANOVA Test; # Tukey HSD; + For patients on catheter drainage; **Qmax** were underleveled as 0, similarly as these patients were not able to recollect their **IPSS**, hence it was not calculated in this group.

**Figure 2:**
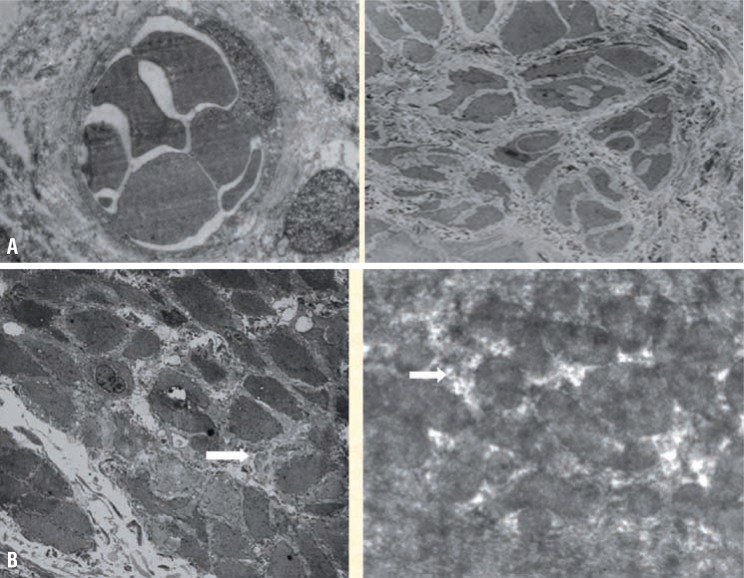
Myohypertrophy (a-X-27600 and b-X-13000). Marked intercellular separation and cellular hypertrophy. Excessive collagen present between the cells as well as between the fascicles. Red arrow shows myohypertrophy and black arrow shows excessive collagen and 2b) shows myohypertrophy in obstructed bladder shown by red arrow and loose fascicle with increased intercellular space shown by black arrow (X-13200).

**Figure 3:**
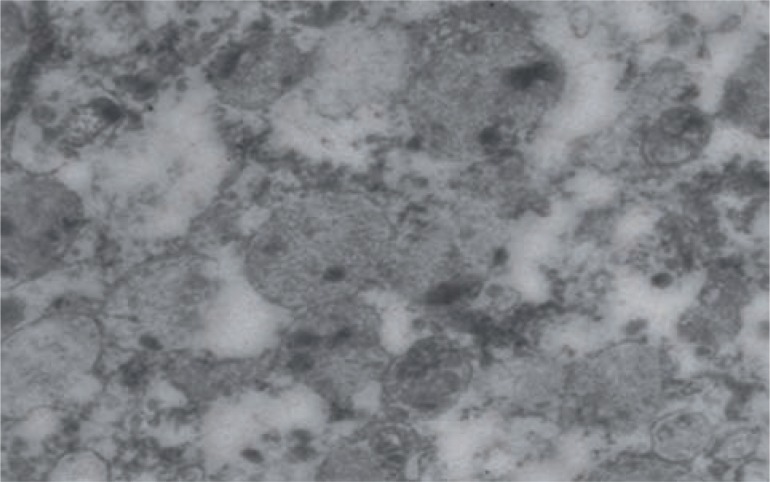
Degenerative pattern (X-12800). Black arrow shows degenerative pattern.

**Figure 4:**
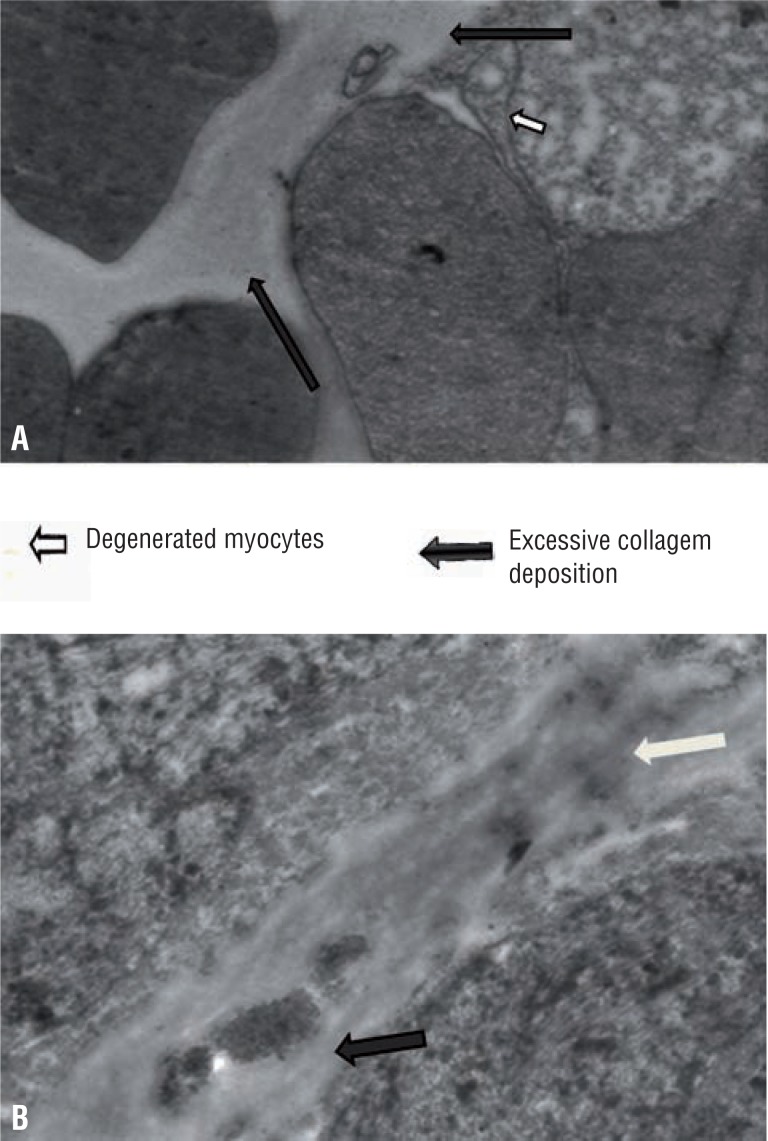
a) Widened intercellular spaces with excessive collagen deposition along with degenerative myocytes (X-25860) and 4b) Excessive collagen with hyperelastosis (X-22380) with black arrow shows collagen and red arrow shows elastin.

**Figure 5:**
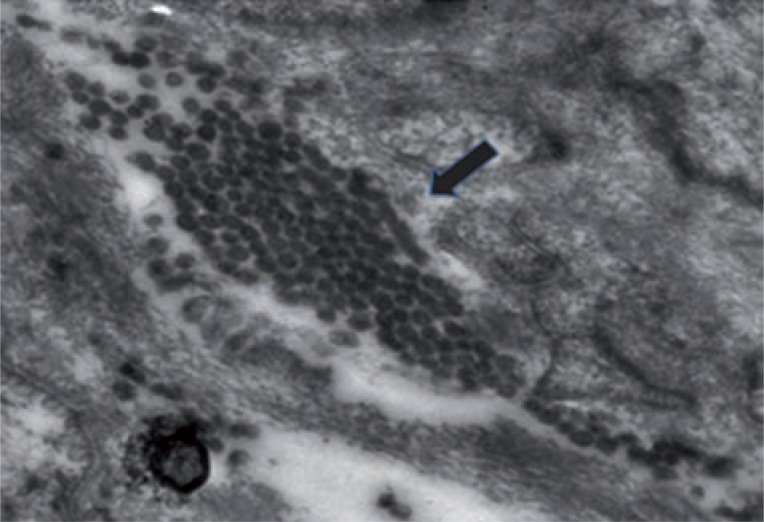
Nerve hypertrophy (X-50220).

Table-2 shows electron microscopic features of control and case group patients. In control group 18 (85.71%) patients had normal myocyte pattern (Figure-1b) whereas majority of patients in case group had varied morphological features. In case group, 9 (31.03%) patients had both myocyte hypertrophy and degenerative pattern (Figure-2a). Degenerative pattern (Figure-3) was seen in 17 (58.62%) case group patients and out of these, 12 (41.37%) patients had either retention of urine or significant PVR before TURP 18 (85.71%) patients of control group had compact fascicular arrangement whereas, in case group loose fascicular pattern was more prevalent which was seen in 19 patients (65.52%) (Figure-2b). Normal interstitial pattern was seen in all control group patients whereas increased collagen (Figure 4a and 4b) was seen as the predominant interstitial pattern in case group in 13 patients (44.83%). Nerve hyper-trophy (Figure-5) was absent in all control group patients while it was present in 23 (79.31%) patients of case group. Normal communication pattern (Figure-1c) was present in all control group patients, whereas in case group 15 (51.72%) patients showed dysjunction pattern.

**Table 2:** Comparison of electron microscopic findings between groups and statistical correlations.

Microscopic findings		Control (Group 1)		Case (Group 2)				Total Case (N=29)	
		(N=21)		Catheter free (A) (N=20)		On catheter (B) (N=9)			
		No.	%	No.	%	No.	%	No.	%
**Myocytes**									
	Normal	18	85.71	4	20.00	1	11.11	5	17.24
	Hypertrophy	3	14.29	5	25.00	2	22.22	7	24.14
	Degeneration	0	0.00	5	25.00	3	33.33	8	27.59
	Hypertrophy+Degeneration	0	0.00	6	30.00	3	33.33	9	31.03
**Fascicles**									
	Compact	18	85.71	4	20.00	2	22.22	6	20.69
	Intermediate	3	14.29	2	10.00	2	22.22	4	13.79
	Loose	0	0.00	14	70.00	5	55.56	19	65.52
**Interstitial tissue**									
	Normal	21	100.00	6	30.00	2	22.22	8	27.59
	Collagen	0	0.00	8	40.00	5	55.56	13	44.83
	Elastin	0	0.00	4	20.00	0	0.00	4	13.79
	Collagen+Elastin	0	0.00	2	10.00	2	22.22	4	13.79
**Nerve hypertrophy**									
	Absent	21	100.00	16	80.00	7	77.78	23	79.31
	Present	0	0.00	4	20.00	2	22.22	6	20.69
**IC Junction (Communication)**									
	Normal	21	100.00	9	45.00	5	55.56	14	48.28
	Dysjunction Pattern	0	0.00	11	55.00	4	44.44	15	51.72

Table-3 shows that on application of chi—square test and Fischer exact test there was a statistically significant difference in all electron microscopic patterns in the control and case groups. However, there was no statistically significant difference in microscopic patterns among the subcategories of the case group.

**Table 3:** Statistical correlation of electron microscopic findings in various groups.

Abnormality of electron microscopic pattern	Control (Group 1) v/s Cases (Group 2)	Control (Group 1) v/s Case (Group 2 A)	Control(Group 1) v/s Case (Group 2 B)	Case A v/s B (Group 2A v/s Group 2B)
**Myocytes** *	<0.001	<0.001	<0.001	1.000
**Fascicles** *	<0.001	<0.001	<0.001	0.643
**Interstitial Tissue** *	<0.001	<0.001	<0.001	0.544
**Nerve Hypertrophy** #	0.033	0.048	0.083	1.000
**IC junction (Communication)** #	<0.001	<0.001	0.005	0.700

* Chi-square Test;

# Fisher Exact Test

Table-4 shows the correlation of IPSS and PVR with all five ultrastructural pattern and it is clear from this Table that if we subcategorize these patients, into IPSS <19 and >19, then although abnormality is more evident in patients with severe symptoms (IPSS >19) it was not statistically significant. Similarly in cases of PVR, ultrastructural characteristics like myocytes and communication pattern are more evident in severe symptoms (PVR >300mL) but these were statistically insignificant.

**Table 4:** Showing correlation of ultrastructural characterstics with IPSS and PVR.

Ultrastructural characterstics		IPSS				PVR			
		<19	>19	Total	‘p’ Value*	<300mL	>300mL	Total	‘p’ Value*
**Myocytes**	Normal	2 (22.22%)	0 (0%)	2	0.099344	5 (22.73%)	0 (0%)	5	0.165377
	Abnormal	7 (77.78%)	11 (100%)	18		17 (77.27%)	7 (100%)	24	
**Fscicles**	Normal	2 (22.22%)	2 (18.18%)	4	0.82218	4 (18.18%)	2 (28.57%)	6	0.555599
	Abnormal	7 (77.78%)	9 (81.82%)	16		18 (81.82%)	5 (71.43%)	23	
**Interstitial tissue**	Normal	3 (33.33%)	3 (27.27%)	6	0.76857	6 (27.27%)	2 (28.57%)	8	0.946613
	Abnormal	6 (66.67%)	8 (72.73%)	14		16 (72.73%)	5 (71.43%)	21	
**Nerve Hypertrophy**	Normal	8 (88.89%)	9 (81.82%)	17	0.65952	17 (77.27%)	6 (85.71%)	23	0.631069
	Abnormal	1 (11.11%)	2 (18.18%)	3		5 (22.73%)	1 (14.29%)	6	
**IC Junction (Communication)**	Normal	4 (44.44%)	6 (54.55%)	10	0.65309	11 (50%)	3 (42.86%)	14	0.741854
	Abnormal	5 (55.56%)	5 (45.45%)	10		11 (50%)	4 (57.14%)	15	

* Chi-square Test

Table-5 shows the postoperative outcome after surgery (TURP). After TURP, all patients except two, successfully voided after catheter removal. In these two patients, catheter was kept for longer duration (15 days), after that one of them was able to void. These patients also had urodynamic finding of hypotonic bladder and complete myocyte degenerative pattern on electron microscopy. On analysis, there was significant improvement in postoperative clinical parameters like IPSS, Qmax, decrease in PVR and prostatic size in the case group when compared to preoperative parameters. However, when compared to control group, the postoperative outcome of case group were found to be inferior.

**Table 5:** Post operative (at 3^rd^ months) comparison between groups.

	Group	N	Mean	Std. Deviation	‘p’ Value*	Significant difference from#
IPSS	Control (Group 1)	21	3.19	1.29		A
	Case (Group 2A)	20	6.65	4.40	0.003	1
	Case (Group 2B) +	8	4.75	1.67		–
Flow Rate(Qmax)	Control (Group 1)	21	17.37	2.45		A,B
	Case (Group 2 A)	20	12.13	4.66	<0.001	1
	Case (Group 2 B)	9	11.36	4.71		1
Prostate Size(in grams)	Control (Group 1)	21	16.00	2.35		A,B
	Case (Group 2 A)	20	24.30	3.85	<0.001	1
	Case (Group 2 B)	9	23.56	4.95		1
PVR (in mL)	Control (Group 1)	21	5.33	5.83		–
	Case (Group 2 A)	20	13.70	26.2	0.061	–
	Case (Group 2 B)	9	59.78	132.17		–

* ANOVA Test;

# Tukey HSD;

+ one patient not able to void

## DISCUSSION

Initial electron microscopic studies suggested that aging can lead to morphological changes in bladder musculature (4, 6, 10). However, recent studies failed to demonstrate these findings (11–13). In our study, we did not find any correlation between morphological changes and aging, as nearly all of our age matched control patients had normal muscular architecture in bladder on electron microscopy. Probably these observational differences among various studies might be due to the variation in selection criteria and lack of control group.

Collado et al. reported that increased outlet resistance may lead to compensatory myohyperpredicting outcome and future treatment trophy of detrusor muscles in bladder outlet obstruction (BOO) (14). These hypertrophied myocytes are responsible for increased collagen and elastin synthesis and deposition in interstitium (11, 14). However, there is no consensus regarding the amount of collagen deposited in interstitium of obstructed bladder (15, 16). Mirone et al. showed that detrusor collagen content increases in symptomatic cases of obstructive BPE and this could be responsible for persistence LUTS (17). Our finding also supported the concept that myohypertrophy is responsible for increased collagen deposition in bladder outlet obstruction (BOO), as nearly all of our case group patients having myohypertrophy also had increased interstitial tissue content. However, whether excessive collagen deposition is more frequently seen in acute (14) or chronic retention (12), is still an unanswered question. Notably, we failed to find any difference in interstitial tissue content among sub-categories of case group.

Various studies had shown that degenerative changes in bladder muscle are responsible for increased residual urine, which persists even after TURP (9). In the present series, all patients having degenerative changes were able to void postoperatively, except two patients, without significant PVR. These two patients were found to have severe degenerative myocytes changes. This suggests that not only presence but severity of degenerative pattern also affects the outcome.

BOO induced growth factors regulate bladder remodeling by different mechanisms (17). The increased expression of nerve growth factor (NGF) is reported to be a key factor in the neuronal hyper-trophy observed in BOO patients (18). In our study, nerve hypertrophy was seen in six patients although individual significance of this feature could not be correlated with any clinical outcome. But we believe that along with muscle hypertrophy, it may be one of the adaptive change in response to outlet obstruction. In the present study, we also found significant correlation between increase in prostatic volume, PVR, IPSS and decrease in Qmax, with the morphological changes in bladder muscle, although this had a nonlinear relationship.

In an animal study, Kim et al. had shown that, changes in detrusor can regress after the correction of obstruction (1). Although there was significant improvement in IPSS, PVR, UFM after TURP in our case group, the outcomes were inferior when compared to control group. These findings suggest that, in contrast to study by Kim et al. (1), these various ultrastructural changes could not revert to normal even after removal of obstruction. That is why patients having severe morphological changes continue to have bothersome IPSS and poor flow rate even after TURP. We believe that probably these patients had detrusor changes which did not revert back to normal state, even after relief of obstruction, and were responsible for persistent symptoms. So, morphological parameter can have a role in predicting outcome and future treatment for bladder dysfunction. Limitation of our study includes less number of patients and lack of post TURP morphological data to confirm the residual detrusor changes. With enrolment of more number of patients and quantification of morphological changes, impact of these changes on various clinical findings could have been better explored.

## CONCLUSIONS

In our study detrusor morphological changes have been seen in various combinations in cases of BOO. These ultrastructural patterns showed nonlinear correlation with clinical measures of bladder dysfunction. Nerve hypertrophy, which was not thoroughly discussed in previous studies, is one of the key features of this study. Surgery definitively leads to improvement in various clinical parameters but these parameters could not reach up to the level of control group because of underlying morphological changes.

## ABBREVIATIONS

BOO: = Bladder outlet obstruction
BPE: = Benign prostatic enlargement
ICJ: = Intercellular junction
IPSS: = International prostate symptom score
LUTS: = Lower urinary tract symptoms
NGF: = Nerve growth factor
PDE 5I: = Phosphodiesterase inhibitors
PSA: = Prostatic specific antigen
PVR: = Post void residual urine
Qmax: = Peak flow rate
TURP: = Transurethral resection of prostate
UDS: = Urodynamics studies
UFM: = Uroflowmetry
USG: = Ultrasonography
